# Physiological responses of Atlantic cod to climate change indicate that coastal ecotypes may be better adapted to tolerate ocean stressors

**DOI:** 10.1038/s41598-024-62700-0

**Published:** 2024-06-05

**Authors:** Diana Perry, Elena Tamarit, Erika Sundell, Michael Axelsson, Sanne Bergman, Albin Gräns, Martin Gullström, Joachim Sturve, Håkan Wennhage

**Affiliations:** 1 Department of Aquatic Resources, Institute of Marine Research, Swedish University of Agricultural Sciences, Lysekil, Sweden; 2 Department of Earth Sciences, University of Gothenburg, Gothenburg, Sweden; 3 Department of Animal Environment and Health, Swedish University of Agricultural Sciences, Gothenburg, Sweden; 4 Department of Biological and Environmental Sciences, University of Gothenburg, Gothenburg, Sweden; 5 The Arctic University Museum of Norway, UiT – the Arctic University of Norway, Tromsø, Norway; 6 School of Natural Sciences, Technology and Environmental Studies, Södertörn University, Huddinge, Sweden

**Keywords:** Metabolism, Climate-change ecology

## Abstract

Healthy ecosystems and species have some degree of resilience to changing conditions, however as the frequency and severity of environmental changes increase, resilience may be diminished or lost. In Sweden, one example of a species with reduced resilience is the Atlantic cod (*Gadus morhua*). This species has been subjected to overfishing, and with additional pressures such as habitat degradation and changing environmental conditions there has been little to no recovery, despite more than a decade of management actions. Given the historical ecological, economical, and cultural significance of cod, it is important to understand how Atlantic cod respond to global climate change to recover and sustainably manage this species in the future. A multi-stressor experiment was conducted to evaluate physiological responses of juvenile cod exposed to warming, ocean acidification, and freshening, changes expected to occur in their nursery habitat. The response to single drivers showed variable effects related to fish biometrics and increased levels of oxidative stress dependent parameters. Importantly, two separate responses were seen within a single treatment for the multi-stressor and freshening groups. These within-treatment differences were correlated to genotype, with the offshore ecotype having a heightened stress response compared to the coastal ecotype, which may be better adapted to tolerate future changes. These results demonstrate that, while Atlantic cod have some tolerance for future changes, ecotypes respond differently, and cumulative effects of multiple stressors may lead to deleterious effects for this important species.

## Introduction

As the climate changes, species must either be able to tolerate the changes, move to more favorable conditions or adapt to the new situation, otherwise they risk facing extinction^1^. Ocean warming and acidification, declining water oxygen levels, freshening (lower salinity), and other escalating environmental drivers affect species tolerance, adaptation, and population dynamics, and can result in ecological regime shifts in marine ecosystems^2–4^. While all species and ecosystems have some natural degree of resilience and adaptability to environmental changes^5^, with the ongoing increased frequency and severity of these pressures, resilience may be diminished or even lost^6^, which will cause stress for both species and ecosystems^7^.

Reduced resilience of marine systems is driven by regional and local scale pressures and may be further exacerbated by interaction with global climate change in the future^8^. If multiple interacting perturbations reduce the resilience of a system, local management may be ineffective on a global scale, but can be effective in reducing some regional effects of climate change, for instance by reducing ocean acidification through local pollution reduction and alkalinization, or by reducing the impacts of sea-level rise through vegetated habitat protection or restoration^9^. Consequently, management strategies focused on smaller scales may become increasingly important for ameliorating the impacts of an inevitably changing environment^10^. At the same time, oceans are experiencing a multitude of changes due to anthropogenic influence which have global scale effects^11^. Human-induced impacts in many areas around the world from e.g. overfishing, ocean and land-based nutrient leaching, the accumulation of toxins, and habitat alteration have led to species die offs, changes in food-web dynamics, and habitat destruction, among other problems, thus creating a marine environment with multiple pressures^12^. As such, marine species are exposed to multiple cumulative drivers which can lead to deleterious effects and a multiple stressor environment^7^.

Atlantic cod (*Gadus morhua*) numbers, for instance, have reached critically low levels in the Baltic Sea^13–15^ and show dramatic declines on the Swedish west coast due to overfishing^16,17^. Additionally, habitat degradation, and reduced marine environmental quality have ultimately left cod populations on the brink of collapse. Despite more than a decade of cod recovery plans for both the North Sea (Council Reg. 423/2004) and the Baltic Sea (2001) and no-take zones established on the west coast^18^, there are little, or no signs of a recovery^14,19–22^. It is becoming increasingly evident that changes other than overfishing (e.g.*,* environmental stressors and altered food-web interactions) must be considered if cod stocks are to recover^23^. This situation for depleted cod stocks becomes even more worrisome given the dramatic alterations expected to occur as a result of global climate change in Swedish waters, such as the freshening, warming (increased temperatures) and ocean acidification (lowered pH)^24–28^. For instance, inshore areas in northern Skagerrak and Kattegat were identified as risk regions for the end of century, with locations showing high risk for potential combined drivers within seagrass ecosystems^29^. This is of importance for juvenile cod within the area, as these vegetated nursery habitats are critical for the species and have strong links to commercial fisheries^30^, with reported effects of loss of seagrass influencing cod recruitment^31^. Additionally, recent genetic evidence suggests that there is a highly heterogeneous cod population system in the North Sea–Skagerrak–Kattegat region and an implication of two distinct ecotypes. Interestingly, both ecotypes, “offshore” and “coastal”, can be found in nearly equal numbers in the Gullmar fjord region where the current study took place^32,33^. Here, the authors postulate that the “coastal” cod ecotype typically performs better in areas with highly stratified salinity gradients, meaning the projected future salinity changes in the region become of particular importance as cod populations more adapted to variability in salinity may be expected to better tolerate freshening conditions. Given the significant decline of Atlantic cod in marine waters surrounding Sweden^34,35^, it is critically important, both ecologically and economically, that any additional potential future threats are well understood in order for management to be able to mitigate effects and avoid a full collapse. This is especially important because juvenile cod are found in many ecologically important habitats along the Swedish coasts^36,37^ connecting coastal and offshore habitats^38–40^.

Understanding the physiological response of cod when potentially stressful environmental conditions, such as temperatures outside their thermal preference, cannot be avoided, is important as ocean changes intensify. Although thermal preference is not constant and varies with life stage, season, environmental conditions such as exposure to hypoxia^41^, and amount of food consumed, it has been shown that juvenile Atlantic cod have a thermal preference range from approximately 8–15 °C depending on hemoglobin type^42^. As such, it is essential that research is done to establish and understand the sensitivity of Atlantic cod to expected future global change drivers, thereby evaluating the cumulative effects of a multiple stressor environment in order to try to protect the future of this important species and manage against population collapse. Laboratory studies help explain the physiological tolerance of fish to the cumulative effects of environmental drivers at specific stages of life. Measures of oxygen consumption rates and oxidative stress are particularly useful for understanding both direct immediate responses and longer term internal changes^43,44^. For instance, fish may respond to a stress initially by altering their rate of respiration, which is reflected by their oxygen consumption rate shown using respirometry, while more long-term chronic exposure to stressors can lead to damages to proteins and increased activities of antioxidant enzymes such as glutathione peroxidase (GPx), glutathione S-transferase (GST), and glutathione reductase (GR)^45,46^. In fact, Kreiss et al.^47^ found that juvenile Atlantic cod exposed to higher temperature (18°C) and ocean acidification show increased oxygen consumption rates compared to those in the lower temperature (10°C) conditions. Changes in antioxidant capacity and cellular damage are used as indicators of long-term exposure to environmental stress^48,49^. An increased metabolism increases reactive oxygen species (ROS) production, and if the generation of ROS exceeds the capacity of defense, oxidative stress leads to severe cellular damage to proteins, lipids, and DNA^50^. This can be a consequence of changes in the aquatic environment, including shifts in temperature, salinity, and pH^51^.

The current study evaluated physiological effects of reduced salinity (freshening), reduced pH (ocean acidification), increased water temperature (warming) and all these stressors combined in juvenile Atlantic cod from the Swedish west coast. These specific environmental drivers have been identified as potentially deleterious for cod and other fish species^52–56^ and are global climate change drivers expected to change within Swedish waters^24,27,57–59^. The aim of this study was to (a) evaluate if oxygen consumption rates and antioxidant defense parameters in juvenile Atlantic cod are affected by these global climate change drivers and if so, (b) determine if cumulative effects can be seen in a multi-stressor environment. The ecotype (coastal or offshore) of half of the fish was also analyzed from genotype sequencing to determine if stress response may be influenced by ecotype. We hypothesized that fish exposed to a greater number of future changes will be more affected as compared to those in a single stressor environment.

## Results

### Summer conditions, marine heatwave

A pilot study of approximately three weeks tested the effects of future summer conditions, specifically exposure to freshening, a marine heatwave, and ocean acidification, and the cumulative effects of all three. For the pilot study, which was conducted in the summer at a water temperature of 21.5 °C and a salinity of 21, there was 100% (20/20) mortality seen in the high temperature treatment and 85% (17/20) mortality in the multi-stressor treatment. There was 30% (6/20) and 20% (4/20) mortality in the low pH and control treatments, respectively, while no deaths occurred in the low salinity treatment (0/20). Significant differences in number of deaths were seen between groups (Kruskal–Wallis H(4, N = 20) = 16.48, *p* = 0.002), with post-hoc pairwise comparisons yielding significant differences between ratio of dead and alive individuals for the low salinity treatment compared to the high temperature and the multi-stressor treatments (*p* = 0.008 and *p* = 0.025, respectively).

### Autumn conditions

#### Fish biometrics

**Figure 1:**
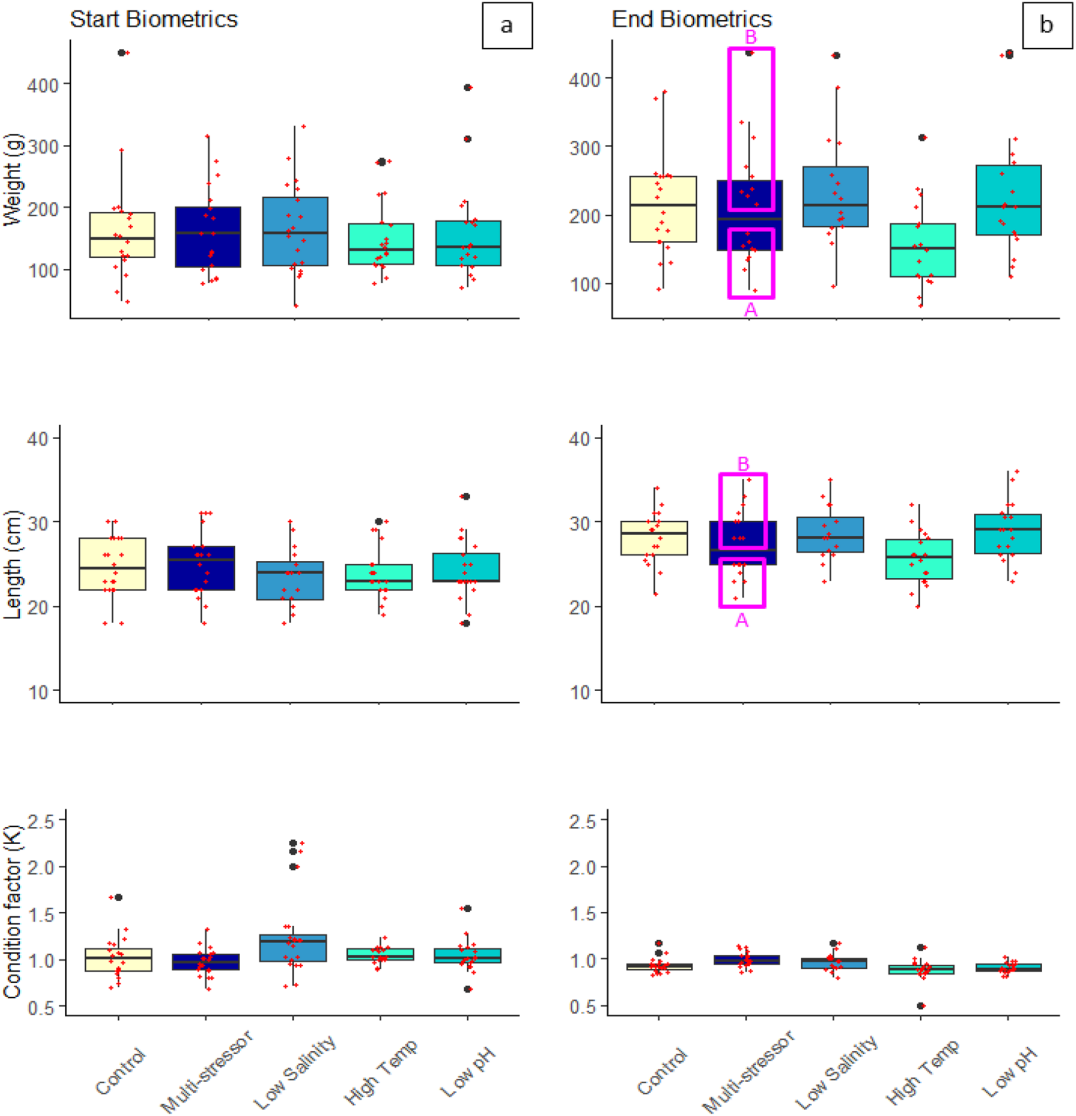
Average weight (g), length (cm), and Fulton’s condition factor (K) of Atlantic cod (*Gadus morhua*) per treatment using box plot graphs with data from each fish scatter plotted (red). The thick solid black line within the boxes indicates the median, with the section above representing the upper quartile, and below the lower quartile. The vertical black line shows the whiskers while the black dots indicate outlier values. Fuchsia boxes indicate two separate groups (A = low, B = high) within the single multi-stressor treatment.

The fish biometrics at the start of the experiment did not differ among treatments for the weight (g) and length (cm) (*p* = 0.99 and *p* = 0.63, respectively (Fig. 1). However the condition factor (K) did differ among treatments (*p* = 0.04) with the post-hoc test showing a significant difference between the multi-stressor treatment and the low salinity treatment (*p* = 0.03) but no treatment differed significantly from the control. Measures of fish biometrics were collected at the end of the four-week experimental exposure period. The weight and length of the fish were taken at the end of the experiment and from that the condition factor (K) was calculated (Fig. 1). Of the 100 fish included in the experiment 10 individuals died throughout the course of the exposure period and were therefore not included in the final measurements for growth, respirometry, or oxidative stress. The control group had no deaths during exposure so n = 20 individuals remained at the end for measurements, the low salinity group had four fish found dead (n = 16 remaining), the high temperature, low pH, and multi-stressor groups had two individuals die during exposure each so n = 18 individuals remained for all three of those treatments. The average final weights differed significantly (*p* = 0.04) with fish in the low salinity group weighing the most at the end of the experiment, while those in the high temperature group weighed the least, also significantly less than those in the low salinity treatment (Table 1). For the average length measurements the treatments differed significantly (*p* = 0.03), and the high temperature group was also the smallest differing statistically from the low pH treatment (Table 1). While the multi-stressor treatment showed the second lowest values both for weight and length, the condition factor (K) was, on the other hand, highest for all groups and significantly different from the high temperature, and low pH groups, with overall condition factor (K) values differing significantly between treatments (*p* = 0.009) (Table 1).

**Table 1:**
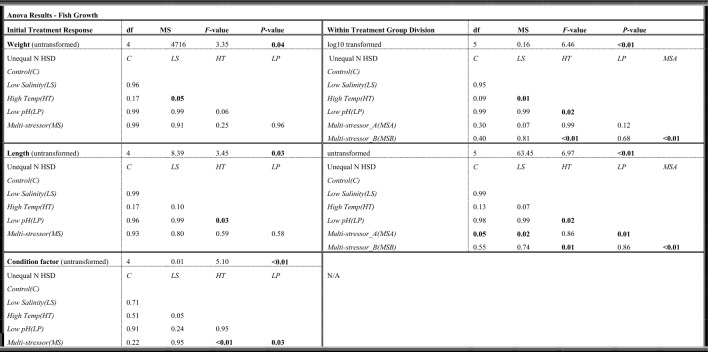
One-way ANOVA and post-hoc results for weight (g), length (cm) and Fulton’s condition factor (K) for comparisons between control, low salinity (freshening), high temperature (warming), low pH (OA), and the multi-stressor treatments (Initial Treatment Response) as well as for comparisons of distinct groups (A and B) within the multi-stressor treatment (Within Treatment Group Division).

Post-hoc *p*-value results shown when ANOVA results were significant (*p* < 0.05) are indicated in bold, with the Unequal N HSD post-hoc test.

The weight and length measurements at the end of the experiment of the multi-stressor treatment indicated that the fish had responded differently to the exposure period. Half of the fish grew considerably more than the other half with two separate groups divided around the median. The upper group was deemed the Multi-stressor_B group and the group below the median the Multi-stressor_A group. A one-way ANOVA showed that differences existed between the treatment groups (*p* < 0.001). The post-hoc analysis revealed statistical differences between the two multi-stressor groups A and B, as well as a difference between Multi-stressor_B and the high temperature treatment, and the high temperature and the low salinity and low pH treatments (Table 1). For the analysis of distinct groups within the multi-stressor treatment regarding length measurements (*p* < 0.001), the Multi-stressor_A group differed significantly from the Multi-stressor_B group as well as the control, low salinity, and low pH treatments. The Multi-stressor_B group also differed from the high temperature treatment, which differed also from the low pH treatment (Table 1).

#### Respirometry

**Figure 2:**
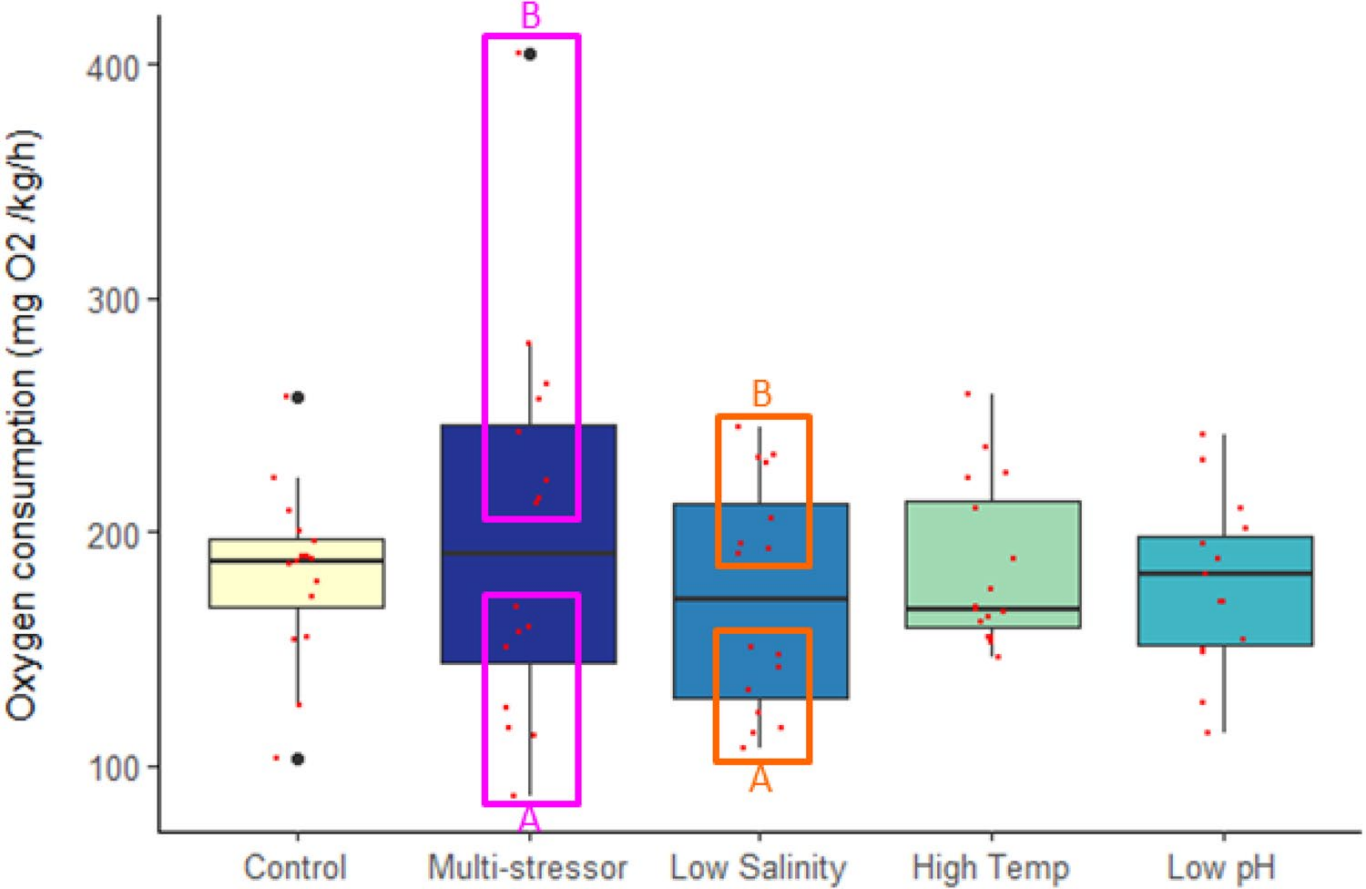
Standard metabolic rate (SMR) in Atlantic cod (*Gadus morhua*) after four weeks of exposure to control conditions, decreased salinity, increased temperature, decreased pH, or a combination of all stressors (multi-stressor). The box plot shows mass-specific MO_2_, with data from each fish scatter plotted (red). The thick solid black line within the boxes indicates the median, with the section above representing the upper quartile, and below the lower quartile. The vertical black line shows the whiskers while the black dots indicate outlier values. Fuchsia boxes indicate two separate groups (A = low, B = high) within the single multi-stressor treatment. Orange boxes indicate two separate groups (A = low, B = high) within the single low salinity treatment.

Respirometry was done to collect oxygen consumption data for the fish at the end of the four week exposure period. The evaluation of the resting mass-specific oxygen consumption (lowest 10% of the fish values generated per fish) averaged per tank showed no significant differences per treatment. The highest average MO_2_ was found in the multi-stressor treatment, while the lowest from the low salinity treatment (Fig. 2).

The respirometry results indicated two distinct groups separated around the median for both the multi-stressor treatment (Multi-stressor_A/B) and the low salinity treatment (Low Salinity_A/B) (*p* < 0.0001). The post-hoc analysis showed the Multi-stressor_B group to be significantly different from all other treatments besides the Low Salinity_B group, with the Multi-stressor_A group differed from the Low Salinity_B group as well as the control, and high temperature treatments (Table 2). The Low Salinity_A group also differed not only from the Low Salinity_B group but also the high temperature and low pH treatments (Table 2).

**Table 2:**
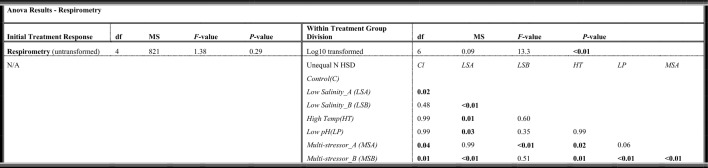
One-way ANOVA and post-hoc results for Atlantic cod (*Gadus morhua*) standard metabolic rate (SMR) (MO_2_) for comparisons between control, low salinity (freshening), high temperature (warming), low pH (OA), and the multi-stressor treatments (Initial Treatment Response) as well as for comparisons of distinct groups (A and B) within the multi-stressor and low salinity treatments (Within Treatment Group Division).

Post-hoc *p*-value results shown when ANOVA results were significant (*p* < 0.05) are indicated in bold.

#### Oxidative stress

**Figure 3:**
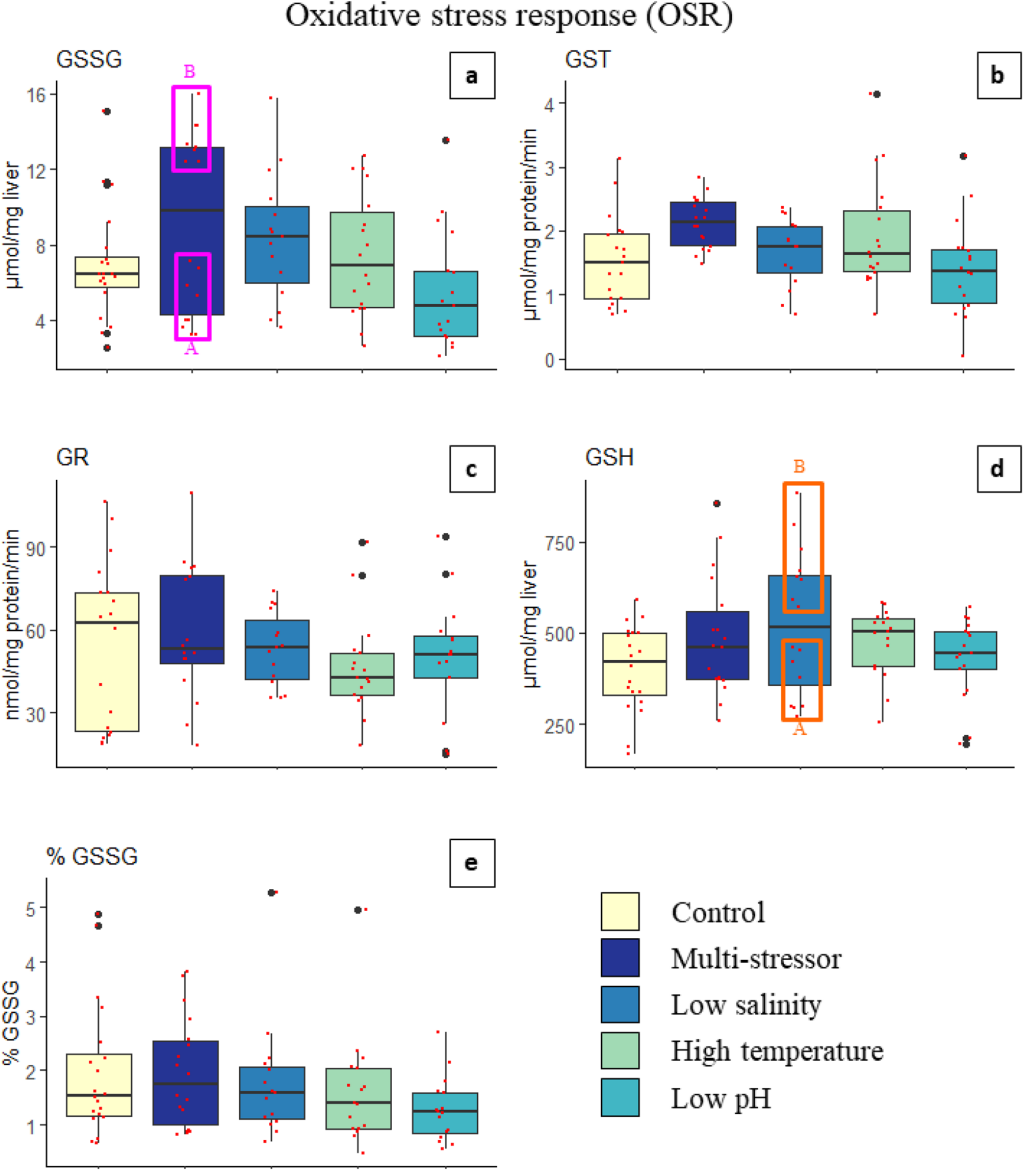
Oxidized glutathione (GSSG), Glutathione-S-transferase (GST), Glutathione-reductase (GR), reduced glutathione (GSH), and percent oxidized glutathione (%GSSG = GSSG/GSH) activity in liver homogenates of juvenile Atlantic cod (*Gadus morhua*) after four weeks exposure to control conditions, decreased salinity, increased temperature, decreased pH, or a combination of all stressors (Multi-stressor). The box plots show enzymatic activity, with data from each fish scatter plotted (red). The thick solid black line within the boxes indicates the median, with the section above representing the upper quartile, and below the lower quartile. The vertical black line shows the whiskers, while the black dots indicate outlier values. Fuchsia boxes indicate two separate groups (A = low, B = high) within a single multi-stressor treatment. Orange boxes indicate two separate groups (A = low, B = high) within a single low salinity treatment.

Antioxidant defense parameters were evaluated at the end of the experiment by collecting the fish livers to analyze enzymatic activity. The mean protein content in cod livers was 5.39 mg ml^−1^ ± 0.40 S.E.M, without any significant differences between treatments (ANOVA; *F* = 1.35, *p* = 0.26). When analyzing for enzymatic activity and glutathione levels in the cod liver homogenates, oxidized glutathione (GSSG) levels and Glutathione-S-transferase (GST) activities showed significant differences between the low pH and multi-stressor treatments (ANOVA; *p* = 0.04 and *p* = 0.01, respectively) (Table 3). The highest mean GST values were found for the multi-stressor treatment. This was followed by the high temperature treatment, and subsequently the low salinity, control, and low pH treatments (Fig. 3). Post-hoc pairwise comparisons showed significant differences in means of GST between the multi-stressor treatment and the low pH treatment.

**Table 3:**
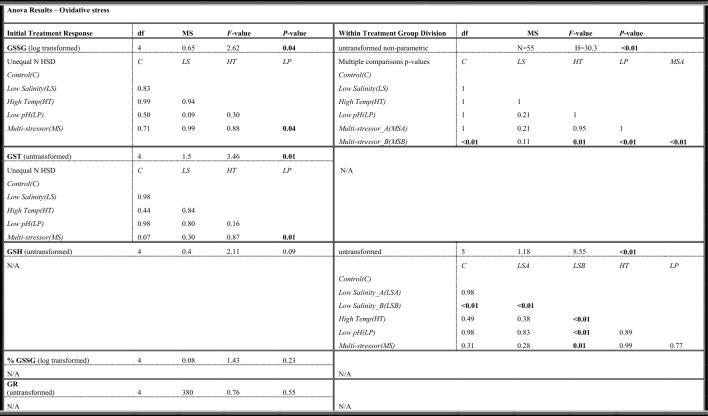
One-way ANOVA and Kruskal–Wallis non-parametric test results for Oxidized glutathione (GSSG), Glutathione-S-transferase (GST), Glutathione-reductase (GR), reduced glutathione (GSH), and percent oxidized glutathione (%GSSG) activity in liver homogenates of juvenile Atlantic cod (*Gadus morhua*) after four weeks exposure to control conditions, decreased salinity, increased temperature, decreased pH, or a combination of all stressors (Multi-stressor) (Initial Treatment Response).

Post-hoc *p*-value results shown when ANOVA results were significant (*p* < 0.05) are indicated in bold. Additional ANOVA results shown for comparisons of distinct groups (A and B) within the Multi-stressor and Low Salinity treatments (Within Treatment Group Division).

GR and GSSG were highest for the multi-stressor treatment and second highest for low salinity (Fig. 3). However, a higher temperature did not increase the mean response of GR and GSSG**.** No significant differences were found in Glutathione-reductase (GR) activities, reduced glutathione (GSH) levels, or percent oxidized glutathione (%GSSG/GSH) between treatments (Fig. 3). Acidification had slightly lower mean values of GSH, GSSG and %GSSG than the control, while freshening had the highest GSH, followed by the multi-stressor and warming treatments.

The oxidative stress analyses yielded two distinct groups in the multi-stressor treatment (A/B) for GSSG (*p* < 0.001) and two distinct groups in the low salinity treatment (A/B) for GSH (*p* < 0.001). The distinct group post-hoc comparisons showed significantly higher values for the Multi-stressor_B group compared to all other treatments, except the low salinity treatment. Additionally, for the GSH post-hoc comparisons, the Low Salinity_B group was significantly higher than all other treatments.

#### Ecotype assignment

**Figure 4:**
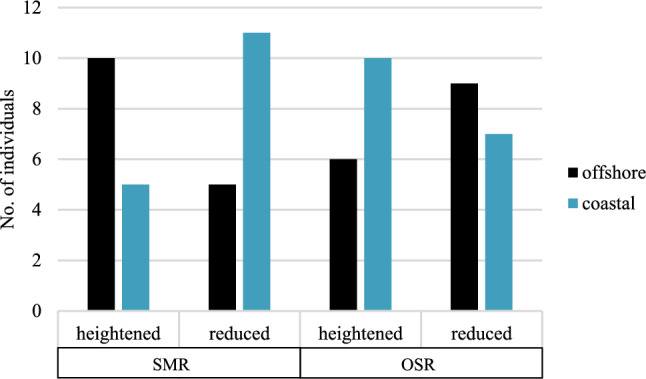
Number of individuals per ecotype (offshore/coastal) with a heightened or reduced standard metabolic rate (SMR) (left) and a heightend or reduced oxidative stress response (OSR) (right) from the within treatment groupings in the low salinity and multi-stressor groups.

DNA samples were genotyped for ecotype diagnostic loci in order to determine if cod ecotype (coastal or offshore) helped explain the within-treatment groupings identified in the physiological response parameters evaluated above. Of the 49 individuals sequenced, 48 samples were valid. In total, there were 20 fish with the offshore ecotype and 28 with the coastal ecotype. From the control treatment there were 5 offshore and 11 coastal ecotype individuals. The multi-stressor and the low salinity treatments were more evenly distributed between ecotypes with 7 offshore and 10 coastal ecotype individuals identified, and 8 offshore and 7 coastal ecotypes and one invalid sample, respectively. To determine whether ecotype was related to the within-treatment variation in stress response, corrected *Chi*-square tests were performed combining the heightened response groups (B) and the reduced response groups (A) for the multi-stressor and low salinity treatments. For the SMR results, there was a significantly higher number of offshore ecotype individuals with a heightened SMR, X^2^ (1, N = 31) = 3.89, *p* = 0.049 (Fig. 4) and coastal ecotype individuals with a low (reduced) rate. Alternatively, no difference was found for ecotype in relation to the oxidative stress within-treatment response, X^2^ (1, N = 32) = 1.13, *p* = 0.288 (Fig. 4). The division of ecotypes per treatment per physiological response is visualized in Supplementary Figures S1, S2 and S3.

## Discussion

This study aimed to understand the physiological effects of a global climate change influenced multi-stressor environment on juvenile Atlantic cod (*Gadus morhua*). Our results indicate that exposure to climate-related stressors influences cod growth and condition factor (K), with warming resulting in reduced growth in terms of both weight and length compared to fish held in control conditions. However, exposure to freshening as well as the multi-stressor environment resulted in significantly higher condition (K) as compared to control conditions. This exciting phenomenon seems to be related to there being two separate responses within these treatments, with half the fish within these treatments showing a heightened stress response and half showing a reduced response for standard metabolic rate and antioxidant parameters. Given these within-treatment divisions in stress response, further analysis was conducted for the fish within these groups to determine whether this could be attributed to genotypic differences. We found, in fact, that two distinct ecotypes within the region were both represented in our study (from the 50% of individuals analyzed) and there was an indication that ecotype plays a role in the physiological responses observed.

Plotting the data revealed that there were in fact two separate oxygen consumptions rate responses within those treatments, one subset with an elevated MO_2_ compared to the controls, and one subset with a reduced respiration rate. A clear relationship has been shown with an increase in metabolic rate as temperature increases for juvenile cod^60^. Additionally, in another experiment looking at the effects of the combination of ocean acidification and temperature on juvenile Atlantic cod it was shown that fish exposed to these combined stressors had a higher standard metabolic rate (SMR)^47^, similar to what we found for the multi-stressor_B group in the current study. This result has been observed for other teleost species, with Atlantic halibut also showing increased metabolic rates as a result of exposure to the combination of warming and ocean acidification^44^. This is particularly interesting given that the current results did not show an effect of these single stressors, warming and ocean acidification, on SMR, but did see a response of freshening as a single stressor. A review of the effects of salinity on a number of other saltwater species revealed similar findings showing that metabolic rate was reduced as salinity decreased from the natural oceanic conditions, which supports the current studies’ results of a significantly decreased SMR for half of the fish within the freshening treatment^61^. However, other species within the review did not show this same pattern and therefore this is not a universal response of saltwater species to osmoregulatory changes^61^. The authors of the review also mention that in the studies evaluated the fish were typically acclimated for greater than 4 days, and that this likely resulted in cellular restructuring associated with the new salinity^61^.

The idea of internal changes occurring as a result of longer term or chronic exposure to stressors has been shown elsewhere with fish species having no respiratory response, however internal physiological changes have occurred^56,62,63^. In contrast however, results from the current study show that within treatment groups for the multi-stressor and the freshening groups significant changes in the standard metabolic rate (SMR) as well as internal antioxidant defense mechanisms were affected. There was a significantly increased amount of oxidized glutathione (GSSG), the oxidized form of the antioxidant glutathione (GSH), for half of the fish exposed to the multi-stressor environment (group B). With the increase in metabolism there is an increase in the production of reactive oxygen species (ROS) and if ROS exceeds the cells capacity for defense oxidative stress can lead to cellular damage of proteins, lipids and DNA^50^. Here, we found that there was an increase in GSH for the within-treatment freshening group (B) as well as an increase in enzymatic activity of GST, an indication that there is oxidative stress occurring, to some degree, for fish exposed to the multi-stressor and freshening conditions. The oxidative stress seen in half of the fish exposed to the multi-stressor and freshening environments as well as the increased SMR, seems to indicate a high degree of plasticity in individual response to stressors. However, there were no clear patterns in those individuals with a heightened SMR and those with an increased level of oxidative stress (i.e. the fish in the within-treatment B groups from the respirometry results in Fig. 2 were not necessarily the same individuals who showed an increased oxidative stress for the within-treatment B groups in Fig. 3). But there was a clear pattern with individuals in the within-treatment differences in growth and the SMR, with the larger individuals showing a lower SMR and smaller individuals having a higher SMR for the multi-stressor group. In other words, we observed that nearly all the individuals in the within-treatment B group for length and weight (Fig. 1) were the same individuals found in the within-treatment group A SMR results (Fig. 2). Such clear patterns in growth and metabolic rate groups were not observed for the freshening treatment. This may be an indication that, while some of the fish within the freshening treatment were clearly showing signs of stress resulting in increased or decreased oxygen consumption as well as oxidative stress, the conditions may not have been as difficult as in the multi-stressor environment where even tertiary effects were observed resulting in changes in growth. This finding follows the studies’ hypothesis that fish in an environment with multiple global climate change stressors will be more greatly affected than those exposed to only single stressors. Although exposure to fresher, lower salinity water, may not have shown effects at all three levels evaluated within the current study (growth, respiration, and oxidative stress), it clearly had an impact, and while it was not significantly higher than the other treatments it was also where the highest amount of mortality was observed, and could perhaps be considered the single dominate driver as has been shown in other studies^64^. From the results of the summer pilot study, however, temperature clearly seems to be the single dominant stressor, which is strong support for the idea that the level of the driver is of critical importance, and that some environmental drivers are not necessarily a stress until a certain threshold is surpassed^7^. The longer term exposure to the marine heatwave values during the summer pilot conditions was clearly so deleterious that the effects of temperature alone were lethal, while if future conditions instead fall within the ranges tested during the autumn conditions experiment, exposure to the multi-stressor environment becomes the most problematic. Although the high mortality rate in both the low salinity treatment under autumn conditions and the MHW treatment under summer conditions supports the idea of a single dominant stressor, the physiological results still support the hypotheses that the cumulative effects of the multi-stressor environment will be more affected overall than those in the single stressor treatments. While single stressors, such as temperature, can have devastating consequences if the stressor passes a certain threshold level, under more long-term lower exposure levels there are a larger degree of physiological changes occurring. From a more evolutionary perspective, a single stressor may produce a selection process which causes a reduction in a portion of the population^65^ but those that survive will be adapted to tolerate the stressor conditions^66^, whereas an environment where many stressors are causing physiological changes on multiple internal levels may be more difficult to adapt to, with little possibility of a selection process occurring.

Exposure to high fishing mortality and stressful environmental conditions for a longer duration can lead to decreased growth affecting the overall fish condition, reproductive potential^67^, and ability to resist disease^68^. This becomes important not only on an individual basis but is relevant on a population wide scale as well given that the mean weight-at-age/length-at-age data is translatable to stock biomass estimates, which are in turn used for calculating fishing quotas and influence management decisions^13^. Larger fish tend to have higher fecundity and many stocks with larger and older individuals have a higher recruitment, while those comprised of smaller individuals who produce fewer eggs and less successful larvae have the potential to have population wide reproductive consequences^69,70^. Clearly, if climate change stressors alter the weight and length development of cod, this may increase predation vulnerability of recruits and lower stock productivity. Additionally, the weight of spawners is positively correlated with recruitment^71^ and therefore reduced growth will lead to consequences for the harvest of fisheries. Much work has been done in understanding how growth of Atlantic cod relates to changes in temperature, with evidence of an acceleration in growth until the optimal temperature is reached and thereafter a rapid decline as temperatures increase^72,73^. Björnsson et al.^72^ even showed that for juvenile cod of a similar weight to those in the current experiment, the optimal temperature for growth was approximately 11–12 °C with clear declines in growth occurring at 16 °C, a result which is supported by the results of the current study showing significantly lower weight and length for the fish exposed to the warming conditions (18 °C). Despite the effects of temperature on growth, another study^73^ showed that Fulton’s condition factor (K) was relatively unperturbed by temperature for fish of the same average size as the current study, similar to the results of the current study showing no difference in K between the warming treatment and that of the controls. Although the relatively good condition of the cod in the multi-stressor and freshening groups compared to the controls is difficult to explain, it is possible that some of the fish in these groups were approaching the limits of their resilience to the stressors, as the within-treatment groups showed some individuals with an increased standard metabolic rate (MO_2_) stress response (Fig. 2), and that if exposure had continued perhaps changes to condition might have been observed.

**Figure 5:**
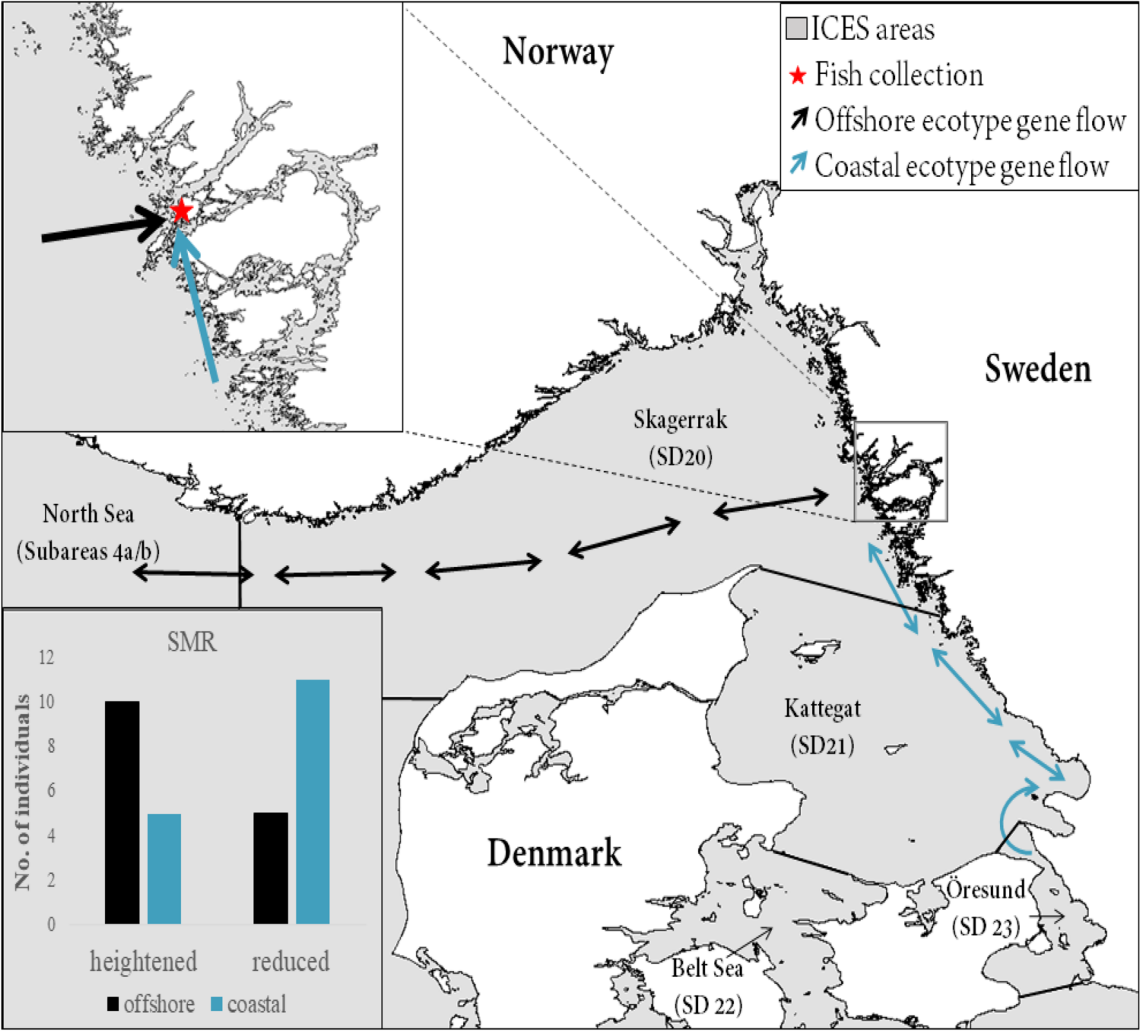
Illustrative map showing the typical genetic origins of the ecotypes (offshore—gold, coastal—blue) of the fish collected (star—red) for experimental exposure. The International Council for the Exploration of the Sea (ICES) subareas and subdivisions (SD) are indicated by a thin black line. The graph showing number of individuals with a heightened or reduced standard metabolic rate (SMR) for each ecotype is in the bottom left corner.

In an attempt to better understand why the current study showed grouped responses, either heightened or reduced, within single treatments fish were genotyped. Although the number of samples were limited and the results somewhat variable, there was an indication that the genetic ecotype of the fish influenced the stress response. Here we found that fish with the offshore ecotype were more often those with a higher SMR, while the coastal ecotype showed a lower or reduced SMR. Studies have shown that juveniles of the coastal ecotype of Atlantic cod in the region are more prevalent at inner-coast locations as well as the Kattegat and Öresund, while offshore cod recruits are found in greater abundance in the North Sea and Skagerrak region, while the area of the current study, Gullmar fjord, has nearly equal numbers of both ecotypes^32,33^. There, the authors point out that coastal areas and locations where coastal cod are found have a more heterogeneous and stratified salinity gradient as compared to offshore areas, and they postulate that the coastal cod may be better adapted to handle the stratified coastal conditions. Our results take this one step further by indicating that the ability to handle the heterogeneous salinity gradients might serve beneficial under future climate change conditions making coastal cod better adapted to handle expected freshening. In such a case, cod stocks from Öresund and Kattegat may have an adaptive advantage to global climate change in Sweden, although further analysis with a larger number of samples is necessary to confirm these indications on a broad scale (Fig. 5).

If so, these results are both critical for management of cod, as well as alarming given the collapsed status of cod in western Sweden and the critically declining cod in the south (Kattegat and Öresund, respectively)^22,74,75^. Additionally, when taken in context of all changes expected due to global climate change additional complications arise given that temperature is expected to continue to increase. Temperature increases will likely drive coastal species farther offshore as coastal areas experience a higher frequency of heatwaves^76^ and possibly even cause range shifts northward as ocean temperatures increase (reviewed by^28^). It was clear from the results of the current studies’ summer conditions that juvenile cod exposed to coastal heatwaves will not survive when unable to move to colder waters, which juvenile cod have been shown to do both for daily shifts in habitats and as more seasonal and ontogenetic shifts^77^. Given the critical importance of coastal shallow-water habitats as nursery areas and refuges from predation a temperature increase causing these habitats to be uninhabitable would have serious consequences for cod recruitment^78^. A behavioral shift in habitats has been clearly shown as a process of temperature avoidance by mobile ectotherms, selecting areas within the temperature preference range of the species in order to cope with thermal stress^79^. However, the latest regional projections indicate that for the Kattegat-Skagerrak area, significant climate change-driven temperature rises are expected for not only surface water, but also for bottom water with proportionally even larger changes expected for bottom water^58^. On top of this, specific areas along the Swedish west coast, particularly in coastal fjord areas, experience periods of hypoxia and even anoxia^80^, a factor with known influence on the growth, reproduction and survivability of cod^81–84^. Thus, the ability of juvenile cod to perform temperature avoidance and shift to cooler habitats is expected to be critically reduced in the region as a result of global climate change and habitat degradation and destruction given that colder deeper waters will be warmer and may no longer be suitable habitats. The present study shows a clear physiological response of juvenile cod to global climate change stressors. The energetic demands of a stressful environment lead to changes in metabolism, liver antioxidant defense, as well as changes in growth. Although the fish response to single stressors varies from no affect to deleterious effects, the response to the multi-stressor environment clearly causes high variability in individual response visible in growth, SMR and OSR even leading to a separation of groups having either a heightened or lowered response. Further evidence of the importance of understanding the response to a multi-stressor environment as single stressor experiments only reveal a portion of the full picture of what to expect under future climate change. Given the myriad of changes the oceans are undergoing and those expected in the future^24–28^, it is of critical importance to understand how keystone species, such as Atlantic cod, respond to the changing environment. Importantly, the current studies’ results indicate that there may be a genetic influence in the degree of physiological response to the environmental changes expected in the future, indicating that the western Baltic and Kattegat stocks in Sweden may actually be better adapted to tolerate aspects of global climate change, a result that requires further investigation. This highlights the need for managing these depleted stocks following the precautionary approach, minimizing all fishing mortality to protect the resilience of this species, and others, to climate change. These findings, in light of the critical status of these cod stocks^14,35,85^, should be considered of dire importance for fisheries management.

## Methods

### Animals

Juvenile cod were collected using wrasse cages from the area around Grundsund (58° 13′ 2.2″ N, 11° 24′ 46.2″ E) on the Swedish west coast (Fig. 5) in October 2020 and transported to the laboratory facilities at the Kristineberg Center (58° 14′ 55.9″ N, 11° 26′ 37.4″ E). A total of 100 juvenile cod (24.3 ± 3.5 cm, 159.4 ± 72.6 g) were used in the experiment. All animal husbandry and experimental conditions were carried out in accordance with relevant guidelines and regulations and were approved by the Swedish Board of Agriculture’s ethical committee in Gothenburg (permit Dnr 83-2016/Dnr 5.8.18-15787/2020). The methods described are reported in accordance with ARRIVE guidelines. The fish were kept in tanks with filtered flow-through sea water pumped in from a depth of 32 m from the ocean outside the laboratory with a 12h:12h light:dark cycle, and were fed daily with a mix of approximately 25–30 g thawed shrimp and herring per aquarium. Fish were observed while feeding and feeding was stopped when interest in the food decreased. Excess food was cleaned from the tanks approximately 1–2 times per week. Fish were held in acclimation at ambient conditions (13.2 °C ± 0.1 SD, 29.7 ± 4.5 SD salinity) for a minimum of 5 days prior to the start of the experimental period.

### Experimental setup and exposure

At the start of the experimental period, all fish were weighed, measured and then randomly placed in one of 20 cylindrical tanks, each with a volume of 3800 L. No differences were found in fish weight or length between treatment groups at the start of the exposure period (weight F(4, 95) = 0.06, *p* = 0.99 average 159.4 g ± 7.3 SE, length F(4, 95) = 0.65, *p* = 0.63 average 24.3 cm ± 0.3 SE). The experimental exposure to different treatments was run for four weeks in November 2020 (“Autumn conditions”) in two thermo-constant rooms at the research facility.

Our experimental design included 5 treatments—control, low salinity (freshening), high temperature (warming), low pH (OA) and the combination of all three stressors (multi-stressor)—with four tanks per treatment and five fish specimens per tank, giving a total of 20 fish per treatment and 100 fish in total. For the purposes of the methods and results, the different treatments will be referred to as control, low salinity, high temp, low pH, and multi-stressor though these are directly related to the global climate change consequences of freshening, warming, and ocean acidification causing a multiple stressor ocean environment. Target experimental values for each treatment were set by taking the last five years of data (2015–2019) from actual water conditions collected from 32 m around the study area at the time of the experiment (https://www.weather.mi.gu.se/kristineberg/en/), removing the highest/lowest 5% of the values in case of potential data errors, and then adding/subtracting the appropriate regional end-of-century values. The end-of-century values were based on the Swedish Meteorological and Hydrological Institute’s (SMHIs) latest regional model predictions, in turn based on the Rossby Centre Atmosphere Ocean model (RCAO) coupled with the Swedish Coastal and Ocean Biogeochemical model (SCOBI)^58,59^. This set our experimental treatment target values at a salinity of 26 (low salinity), a temperature of 18 °C (high temp), and a pH of 7.5 (low pH). For each treatment (except for the multi-stressor treatment), the two other water parameters were kept at ambient conditions, e.g.*,* the salinity treated group received water with a salinity of 26 (manipulated according to future predictions), a temperature of 13 °C and a pH of approximately 8.1. The temperature was increased by approximately 1 °C per day so that by 5 days into exposure temperature had reached the target treatment value (high temperature and multi-stressor treatments). The salinity and pH values were set prior to the experimental start (i.e. not decreased gradually) so exposure to target values began on day one of the experimental exposure period. The control treatment tanks were kept at ambient conditions, with a salinity of 33, a temperature of 13 °C, and a pH of 8.1 (Supplementary Table 1). For the multi-stressor treatment, the tanks were exposed to all three global climate change drivers (freshening, warming and ocean acidification) simultaneously for the full duration of the experimental period. For the low salinity treatment, freshwater was centrally mixed into the seawater input prior to entering the aquaria with a freshwater line added into the main seawater line for the experimental room. This ensured a well-mixed, stable salinity for the water entering each aquarium. For the high temperature treatment, the temperature was centrally controlled by computers set up for the thermo-constant rooms, allowing for two separate temperatures to be maintained. The low pH treatment had pure CO_2_ bubbled into the tanks which mixed as a result of the water circulation due to the aquariums’ flow-through system using a feedback pH–stat computer system (Aqua Medic, Bissendorf, Germany) to maintain a reduced pH of 7.5.

Daily observations of fish and tank conditions were recorded to ensure all equipment was functioning properly. To monitor the conditions throughout the experiment, a number of parameters were measured regularly (1–3 times per week for all tanks), including pH, temperature, salinity, and oxygen saturation using a WTW Multi 3430 pH meter. Note that one or two aquaria were checked approximately daily, and because the salinity and temperature were centrally regulated the aquaria in these treatments varied by a maximum of approximately 0.2 °C and 0.2 salinity unites from the others and thus checking one aquarium was sufficient to see that target values were being maintained. Additionally, water chemistry conditions were recorded once a week, and subsequently water samples were collected for alkalinity measurements using the TA05 plus/TW alpha plus, SI Analytics, (Mainz, Germany) machine. All samples were filtered using a 0.45-lm filter and re-measured for total pH (pH_TS_) using a pH meter calibrated with TRIS (Tris/HCl) and AMP (2-aminopyridine/HCl) buffer solutions with a salinity of 32, after which point samples were placed in the alkalinity machine for total alkalinity (TA) readings. The pH total scale calculation was made using the linear regression curve calculated from the TRIS calibrations. Carbonate system parameters were then calculated from pH_TS_ and TA. The ambient pH groups differed significantly from the low pH treatments (t(98) = 10, *p* < 0.0001). The ambient pH groups had a pH_TS_ of 8.06 ± 0.08 (*p*CO2 of 404 ± 92 μatm), while the low pH treatments had a pH_TS_ of 7.67 ± 0.30 (*p*CO2 of 1439 ± 1692 μatm) and all treatments were supersaturated for both calcite and aragonite (Table 4).

**Table 4:** Carbonate chemistry water measurements taken weekly throughout the 4-week experiment.

Parameter	Ambient pH		Low pH		Control °C		High °C		Ambient Sal		Low Sal		F-ratio var.	*p*-value
	n	mean ± SD	n	mean ± SD	n	mean ± SD	n	mean ± SD	n	mean ± SD	n	mean ± SD		
Alkalinity	60	2308 ± 184	40	2235 ± 217	–	–	–	–	–	–	–	–	1.4	0.08
pH	60	8.06 ± 0.08	40	7.67 ± 0.30	–	–	–	–	–	–	––	–	14.8	< 0.001
*p*CO_2_	60	404 ± 92	40	1439 ± 1692	–	–	–	–	–	–	–	–	339	< 0.001
Temp	–	–	–	–	60	12.95 ± 0.35	40	16.90 ± 0.77	–	–	–	–	4.9	< 0.001
Salinity	–	–	–	–	–	–	–	–	60	32.62 ± 1.92	40	26.39 ± 1.64	1.4	< 0.001

The seawater pH is calculated on the pH total scale.

Dashes indicate values not analyzed.

#### Summer conditions pilot study

An initial pilot study was conducted in August 2020 with flow-through water from 7 m depth from the ocean outside the research station. The study was done to evaluate the intended future target values, test the most suitable duration of exposure of the experiment, and determine if the experiment could be run using maximum summer temperature conditions. The general design was similar to that mentioned above, with the same five treatments and twenty juvenile fish specimens per treatment. Treatment target values, however, reflected maximum summer conditions with a salinity of 21, a temperature of 21.5 °C, and the target pH of 7.5 (which was the same for both study runs). Control conditions were ambient salinity, ambient pH and a temperature of 16 °C (Supplementary Table 1). The intended exposure was four weeks; however, after two and a half weeks high mortality was seen in the high temperature and multi-stressor treatments, and so the study was terminated at approximately three and a half weeks exposure. Due to the premature mortality no respirometry or liver sample analyses were performed.

### Respirometry

Fish from the same treatment group were netted, placed into buckets, and transported directly to the room for respirometry experiments. Each fish was placed into cylindrical transparent respirometers (with a total volume of 3 or 3.9 L depending on fish size) for intermittent flow-through respirometry. The respirometers were submerged into two tanks containing 4 respirometers each. So for each measurement period 8 respirometers (i.e. 8 fish) were run, and each treatment had two measurement periods for a total of 16 fish measured using respirometry per treatment. Each tank continuously received aerated water pumped from the outside ocean and the temperature, pH, and salinity of the water were adjusted according to the specific treatment groups (i.e., freshening, warming, ocean acidification, multi-stressor or control). The fish were kept in the respirometers for 20 h and thereafter euthanized with a sharp blow to the head. After euthanization, weight and length were noted before the fish livers were sampled by sharp dissection. The livers were stored at − 80 °C until further analysis (*see liver oxidative stress analysis*).

#### Respirometry data acquisition and calculations

Each respirometer was connected to an individual recirculation pump and a common flush pump. The recirculation pump constantly recirculated the water within the respirometer and the in- and outflows were connected diagonally to ensure good mixing of the water. An oxygen optode (OXROB, PyroScience, Aachen, Germany) was placed in the outflow of the recirculation loop and a FirestingO_2_ system (FSO2-4, PyroScience, Aachen, Germany) continuously recorded the partial pressure of oxygen within the water. Prior to each respirometry trial, the optodes were two-point calibrated using vigorous bubbling to achieve 100% oxygen saturation, and sodium sulfite to achieve 0% oxygen saturation. The flush pump continuously flushed the respirometers with new, oxygen-saturated water when turned on. Every 20 min the flush pump turned off for five minutes, during which time the oxygen consumption rate of the fish (MO_2_) was calculated by recording the decline in oxygen saturation within the respirometer (the 20 min cycle consisted of a 15 min flush period plus a 5 min measuring period). Background respiration, i.e., the respiration in the respirometer made by bacteria, was measured for 1 h prior to the fish entering the respirometer and for 1 h after the fish exited the respirometer. The calculated oxygen consumption rate from these measurements was used to correct the fish MO_2_ for potential background respiration. MO_2_ (mg O_2_ kg^−1^ h^−1^) was calculated using Eq. 1.

1 $${\text{MO}}_{{2}} = ([\left( {{\text{V}}_{{\text{r}}} - {\text{V}}_{{\text{f}}} } \right) * \Delta {\text{C}}_{{{\text{wO2}}}} ]\Delta {\text{t}}) - ([{\text{V}}_{{\text{r}}} * \Delta {\text{C}}_{{{\text{wO2}}}} ]\Delta {\text{t}})$$

where V_r_ is the volume (L) of the respirometer, V_f_ is the volume of the fish (derived from the body mass [g] of the fish assuming that the density is 1 g ml of tissue^−1^), ΔC_wO2_ is the change in oxygen concentration (% s^−1^) in the water (derived from the measured partial pressure of oxygen, taking temperature and salinity into account) and Δt is the time during which ΔC_wO2_ was calculated. Typically, 60–63 recordings of ΔC_wO2_ were obtained during the 20 h in the respirometers, which resulted in 60 calculated mass specific MO_2_ values per fish.

The output from the Firesting O_2_ system was relayed to a 16SP PowerLab (ADInstrument, Castle hill, Australia) connected to a computer. The computer contained the data acquisition software LabChart pro (7.3.2; ADInstrument, Castle hill, Australia) that, besides acquiring the data, allowed for an automatic on and off switch for the flush pump.

### Liver oxidative stress analysis

#### Antioxidant enzymes

Sample preparation. Liver samples were homogenized (glass/teflon) in 4 volumes (w/v) in ice-cold buffer saline (0.1 M Na/K-PO4), containing 0.15 M KCl at pH 7.4. Homogenates were centrifuged at 10,000 g for 20 min at 4 °C. The supernatants (S9 fraction) were aliquoted and stored at − 80 °C until analysis.

Biochemical analysis. The accumulation of product or consumption of substrate was monitored spectrophotometrically over time on a microplate reader (Spectra Max 190, 96 well plate) at room temperature (20 °C). Enzyme activity was normalized to protein content. Total protein content in the S9 fraction was measured according to Lowry et al. (1951) using bovine serum albumin as standard.

GST activity was measured according to^86^ adapted to a microplate reader according to^87^. Reaction mixture contained 2 mM CDNB, 1 mM GSH in 0.1 M Na phosphate buffer (pH 7.5). Change in absorbance was monitored at 340 nm and the extinction coefficient for glutathione-DNB adduct ε = 9600 M^−1^ cm^−1^ was used to calculate enzymatic activity.

GR activity was measured in liver cytosol according to the method described by^88^. Reaction mixture contained 0.08 mM DTNB and 0.63 mM NADPH in 0.1 M sodium phosphate buffer (pH 7.5) containing 1 mM ethylenediaminetetraacetic acid. After 2 min, the reaction was initiated by the addition of 10 μL of 3.25 mM oxidized glutathione, and the reduction of DTNB was monitored spectrophotometrically at 405 nm. The activity was calculated using the extinction coefficient of TNB (ϵ = 14,151/M/cm).

#### Glutathione levels

Sample preparation. Liver samples were homogenized 4 times in 5% SSA (w/v) with sonication 3 × 3 s, precipitated on ice for 15 min, and centrifuged at 4 °C and 10,000 g for 20 min and supernatant collected.

GSH and GSSG levels were measured independently with an indirect biochemical assay based on the methods of^89^ and adapted to a plate reader by^90^. A reaction mixture containing 10 mM DTNB (tGSH) or 1 mM DTNB (GSSG) and 2 mM NADPH dissolved in a stock buffer (143 mM NaH2PO4 + 6.3 mM EDTA, pH 7.4) was added to wells containing 20 µl of sample. Samples for tGSH were diluted 80 times in 1.3% SSA and samples for GSSG were undiluted. Prior to GSSG analysis reduced GSH was precipitated with vinyl pyridine (5µL/100µL sample) with shaking at RT for one hour. The absorbance was measured at 415 nm and compared to a standard curve with GSH.

### DNA analysis

Fin clip samples were taken from the anal fin of each fish after the fish was removed from the respirometry chambers and terminated (some individuals were not used for respirometry and therefore samples were collected directly after termination). Once the fish were killed, they were put directly on ice until samples were collected (within 30 min or less). The samples were then stored in ethanol in 2 mL microcentrifuge tubes at minus 4 °C until analysis at the Tjärnö laboratory, University of Gothenburg. A total of 49 individuals from the control (n = 16), low salinity (n = 16) and multi-stressor (n = 17) treatments were genotyped for ecotype-diagnostic loci. Ecotype assignment was determined following the methodology used in Henriksson et al.^33^. Note that ecotype assignment was performed as a means of further understanding the within-treatment group separations (Figs. 1, 2 and 3) seen in the low salinity and multi-stressor treatments, and due to logistical limitations the number of samples were restricted. Therefore, the high temperature and low pH treatments were not prioritized for this analysis.

### Data analyses

Graphic visualizations were performed using R Statistical Software (v 4.2.2, R Core Team 2022) in RStudio with the package ggplot2 (Wickham, 2016), Excel and ArcMap (v. 10.5). Statistical analyses were performed using STATISTICA 64 version 13 and R version 4.2.2 (R Core Team, 2022). Data were checked for normal distribution prior to analysis using the Shapiro–Wilk test and for homogeneity of variances using Levene’s (1960) test. When necessary, the data were transformed using square root, log(x) or log_10_(x + 1), and if the data remained heteroscedastic even after transformations, the non-parametric Kruskal–Wallis test was performed. Potential tank effects were tested using nested ANOVAs which found no differences between the tanks in each treatment. One-way ANOVA or Kruskal–Wallis tests were used to determine if significant differences occurred between treatments for weight (g), length (cm), Fulton’s condition factor (K), oxygen consumption rate (MO_2_), and oxidative stress enzymatic activity (Tables 2, 3, and 4). When ANOVA tests showed significant differences between treatments, a posteriori comparisons of means were conducted (Fisher LSD, Tukey unequal means HSD used to account for unequal sample sizes, or non-parametric multiple comparisons *p*-values).

Within treatment, data were checked for deviations around the median, and any treatment that contained the next closest two data points above and below the median, both laying one or more standard error points from the median, were considered two distinct groups within a treatment (where within treatment groups are labeled as A and B, see Figs. 1, 2, and 3 for examples of distinct groups within a treatment). The standard metabolic rate was calculated from the lowest 10% of the values generated under the 20 h measurement period averaged. Outliers were removed (where outliers were measurements of  > 2 standard deviations below the mean of the lowest 10%)^56^. Fulton’s condition factor (K) was calculated using Eq. 2 where W is weight and L is length^91^.

2 $$K=100*\frac{W}{{L}^{3}}$$

From the DNA results, the relationship between offshore and coastal ecotypes and a heightened or reduced stress response was analyzed using the *Chi*-square test (X-square).

### Supplementary Information

Supplementary Information.

## Data Availability

The physiology datasets generated during the current study are available from the corresponding author on reasonable request. Additionally, all the DNA data presented in this study are available at the data repository Zenodo: 10.5281/zenodo.10075426
