# Supplementary material for: Physiological responses of Atlantic cod to climate change indicate that coastal ecotypes may be better adapted to tolerate ocean stressors
Source: Sci Rep. 2024 Jun 5;14:12896. doi: 10.1038/s41598-024-62700-0 (PMC11153577; doi:10.1038/s41598-024-62700-0)
Supplement: Supplementary file 1 — Supplementary Information. [file 41598_2024_62700_MOESM1_ESM.docx]

# Physiological responses of Atlantic cod to climate change indicate that coastal ecotypes may be better adapted to tolerate ocean stressors

Diana **Perry^1*^**, Elena Tamarit^2^, Erika Sundell^3^, Michael Axelsson^4^, Sanne Bergman^5^, Albin Gräns^3^, Martin Gullström^6^, Joachim Sturve^4^ and Håkan Wennhage^1^

**Supplementary Material**

## Results

Graphs showing the genetic ecotype for each analyzed fish (n=48) per treatment for the final fish biometrics (SFigure 1), standard metabolic rate (SFigure2) and the oxidative stress (SFigure 3).


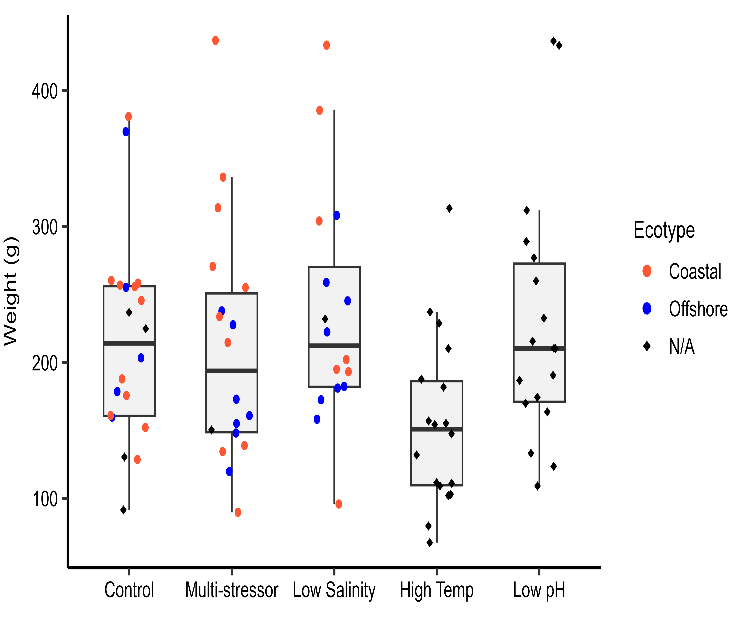

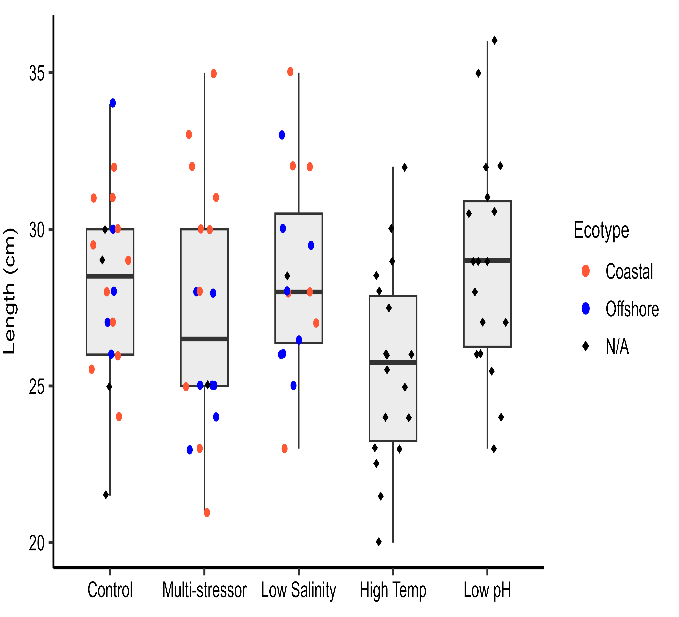

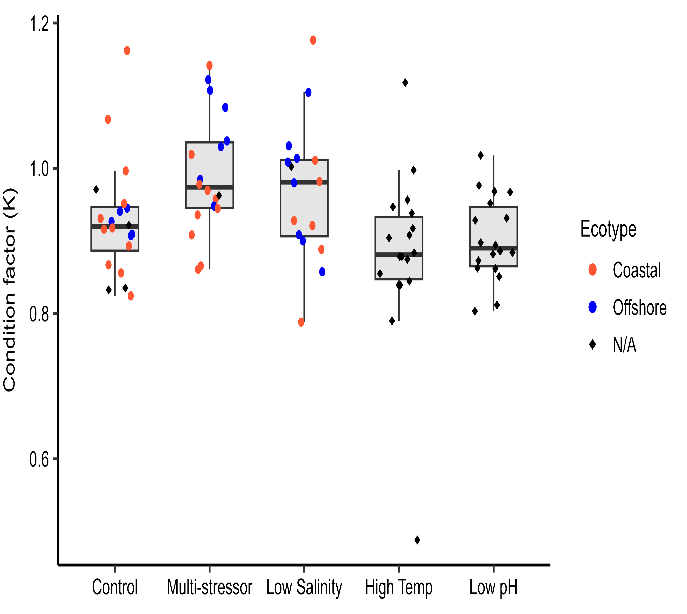


SFigure 1: Weight (g), length (cm), and Fulton’s condition factor (K) of Atlantic cod (*Gadus morhua*) per treatment at the end of the experiment with genetic ecotype shown per fish (offshore – blue, coastal- orange, or not analyzed – black).


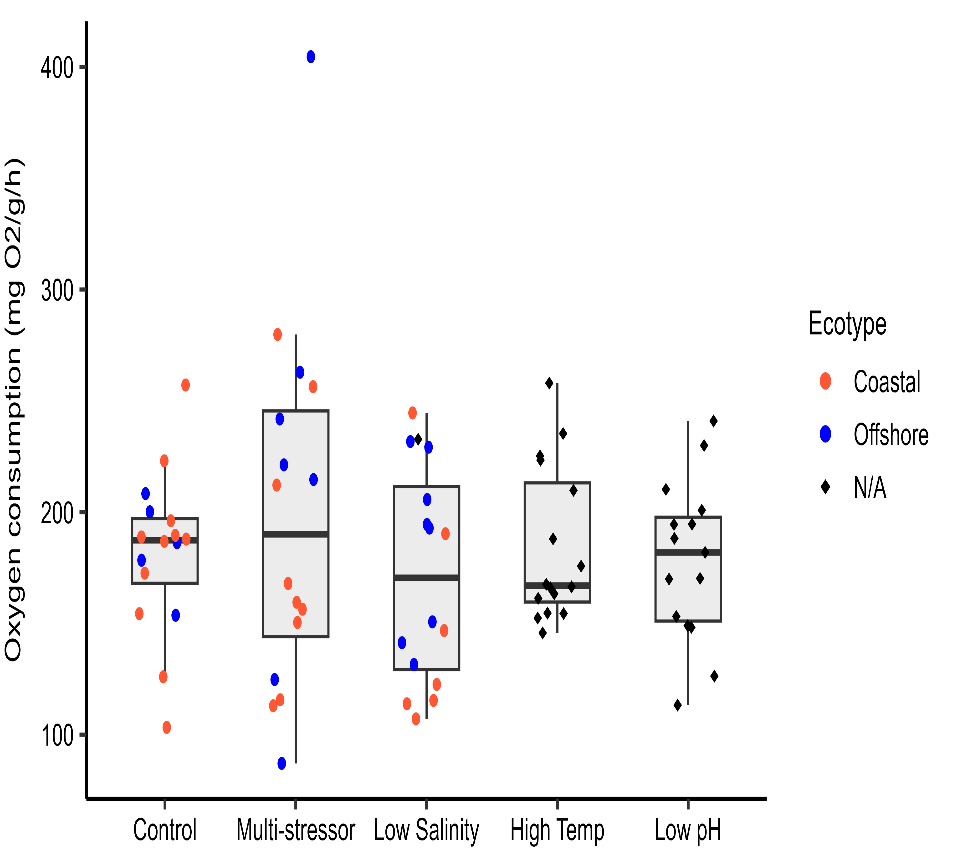


SFigure 2: Standard metabolic rate (SMR) in Atlantic cod (*Gadus morhua*) after exposure to control conditions, decreased salinity, increased temperature, decreased pH, or a combination of all stressors (Multi-stressor) with genetic ecotype shown per fish (offshore – blue, coastal- orange, or not analyzed – black).


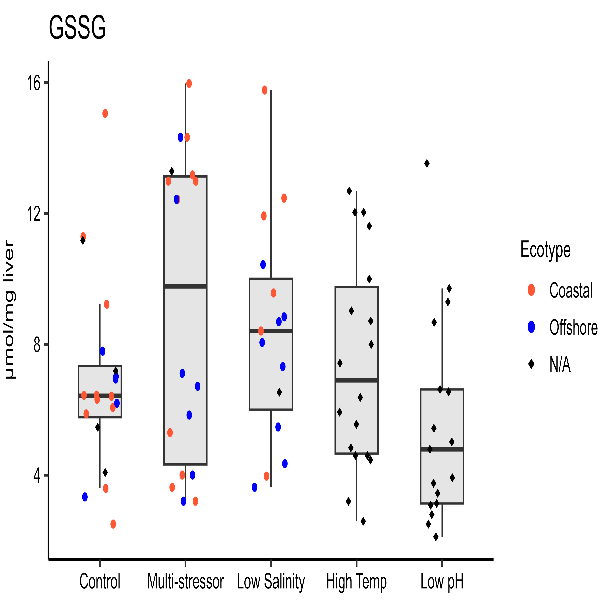

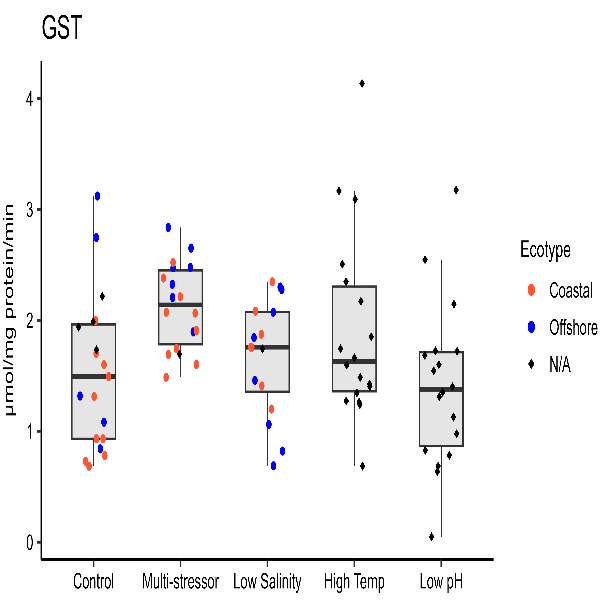

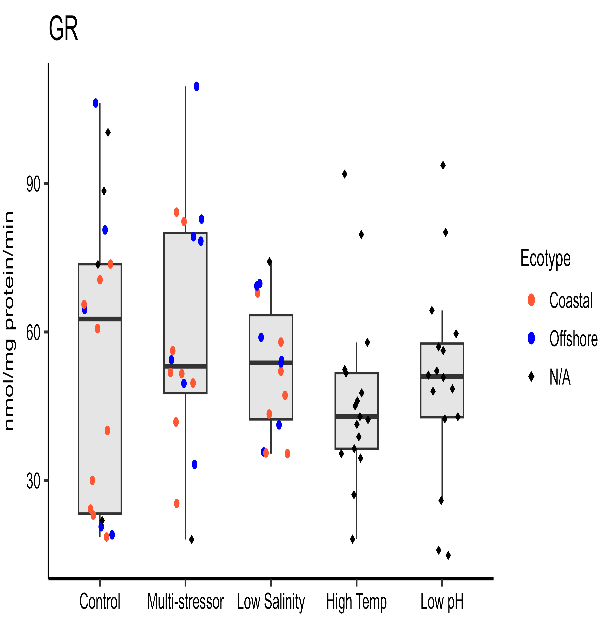

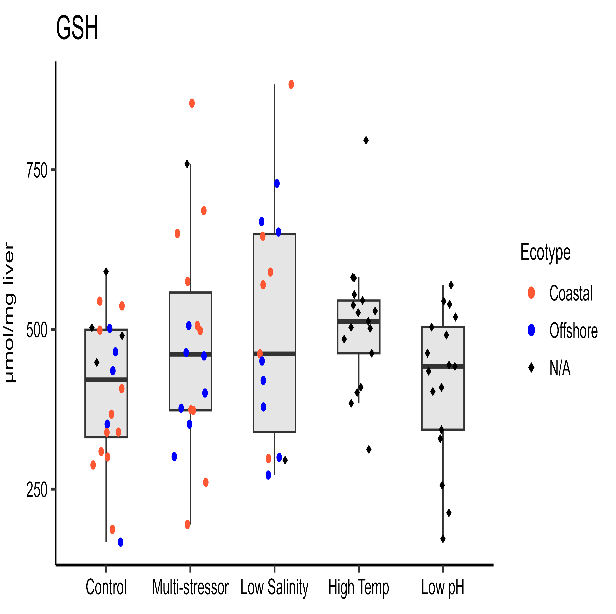

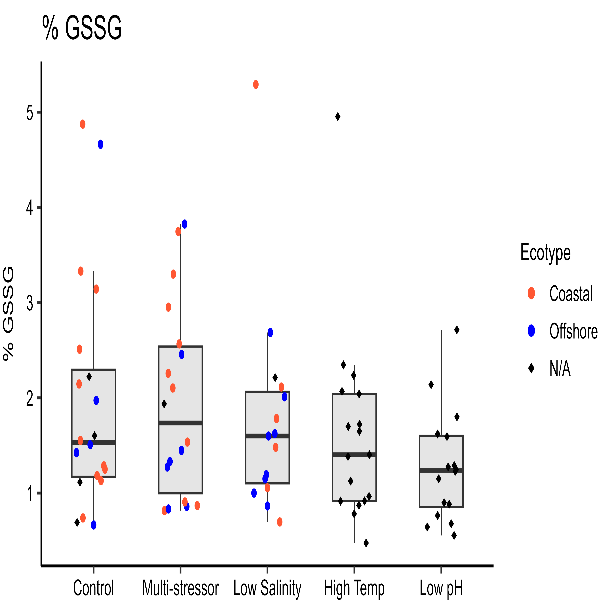


SFigure 3: Oxidative stress response measured by analysis of Oxidized glutathione (GSSG), Glutathione-S-transferase (GST), Glutathione-reductase (GR), reduced glutathione (GSH), and percent oxidized glutathione (%GSSG =GSSG/GSH) activity in liver homogenates of juvenile Atlantic cod (*Gadus morhua*) after exposure to control conditions, decreased salinity, increased temperature, decreased pH, or a combination of all stressors (Multi-stressor) with genetic ecotype shown per fish (offshore – blue, coastal- orange, or not analyzed – black).

## Methods

List of chemicals used for the liver oxidative stress analysis

**Chemicals**. β-Nicotinamide adenine dinucleotide 2′-phosphate reduced tetrasodium salt hydrate (NADPH), oxidized L-Glutathione (GSSG), L-Glutathione reduced (GSH), Glutathione reductase (GR), 2-vinylpyridine, 5.5’- Dithio-bis 2-Nitrobenzoic Acid (DTNB) and 1-Chloro-2,4-dinitrobenzene (CDNB) were obtained from Sigma Aldrich (St. Louis, MO, USA). (Ethylenedinitrilo)tetraacetic acid (EDTA) and salts were of analytical grade and purchased from Merck.

STable 1: Mean water condition values per treatment during the summer pilot study with an approximately 3 week exposure period.

| **Treatment** | **Salinity**  **mean ± SD** | **Temp (°C)**  **mean ± SD** | **pH**  **mean ± SD** | **Dissolved O_2_ (mg/L) mean ± SD** | **Alkalinity (µmol kg −1) mean ± SD** |
| --- | --- | --- | --- | --- | --- |
| Control | \| 26.76 \| (3.50) \| \| --- \| --- \| | \| 16.39 \| (0.62) \| \| --- \| --- \| | \| 7.92 \| (0.23) \| \| --- \| --- \| | \| 9.95 \| (0.34) \| \| --- \| --- \| | \| 2198.25 \| (311.70) \| \| --- \| --- \| |
| Mulit-stressor | \| 21.25 \| (1.29) \| \| --- \| --- \| | \| 20.56 \| (0.78) \| \| --- \| --- \| | \| 7.60 \| (0.29) \| \| --- \| --- \| | \| 9.75 \| (0.40) \| \| --- \| --- \| | \| 1891.50 \| (419.67) \| \| --- \| --- \| |
| Low Salinity | \| 21.63 \| (0.79) \| \| --- \| --- \| | \| 16.44 \| (0.35) \| \| --- \| --- \| | \| 8.04 \| (0.13) \| \| --- \| --- \| | \| 9.94 \| (0.30) \| \| --- \| --- \| | \| 1923.00 \| (49.28) \| \| --- \| --- \| |
| High Temp | \| 27.81 \| (3.91) \| \| --- \| --- \| | \| 21.49 \| (2.30) \| \| --- \| --- \| | \| 8.07 \| (0.11) \| \| --- \| --- \| | \| 9.58 \| (0.45) \| \| --- \| --- \| | \| 2204.46 \| (120.13) \| \| --- \| --- \| |
| Low pH | \| 25.35 \| (3.78) \| \| --- \| --- \| | \| 16.14 \| (0.23) \| \| --- \| --- \| | \| 7.58 \| (0.24) \| \| --- \| --- \| | \| 9.96 \| (0.43) \| \| --- \| --- \| | \| 2156.34 \| (186.75) \| \| --- \| --- \| |
